# Spatial-Temporal Congestion Identification Based on Time Series Similarity Considering Missing Data

**DOI:** 10.1371/journal.pone.0162043

**Published:** 2016-09-20

**Authors:** Hongsheng Qi, Meiqi Liu, Dianhai Wang, Mengwei Chen

**Affiliations:** College of Civil Engineering and Architecture, Zhejiang University, 866, Yuhangtang Road, Hangzhou, 310058, China; Beihang University, CHINA

## Abstract

Traffic congestion varies spatially and temporally. The observation of the formation, propagation and dispersion of network traffic congestion can lead to insights about the network performance, the bottleneck dynamics etc. While many researchers use the traffic flow data to reconstruct the congestion profile, the data missing problem is bypassed. Current methods either omit the missing data or supplement the missing part by average etc. Great error may be introduced during these processes. Rather than simply discarding the missing data, this research regards the data missing event as a result of either the severe congestion which prevent the floating vehicle from entering the congested area, or a type of feature of the resulting traffic flow time series. Hence a new traffic flow operational index time series similarity measurement is expected to be established as a basis of identifying the dynamic network bottleneck. The method first measures the traffic flow operational similarity between pairs of neighboring links, and then the similarity results are used to cluster the spatial-temporal congestion. In order to get the similarity under missing data condition, the measurement is implemented in a two-stage manner: firstly the so called first order similarity is calculated given that the traffic flow variables are bounded both upside and downside; then the first order similarity is aggregated to generate the second order similarity as the output. We implement the method on part of the real-world road network; the results generated are not only consistent with empirical observation, but also provide useful insights.

## Introduction

### Background

As the number of vehicles steadily increases and the expansion of roadways is relatively slow, traffic congestion is becoming a central transportation issue in big cities. The traffic congestion is a result of the imbalance between traffic supply and demand. The bottleneck is the weak point of the supply-demand structure. Thus generally congestion first begins from these bottlenecks and then propagates around the network. Sometimes the process is called cascading failures [1,2]. Such daily road network operation can be observed experimentally from traffic flow data. Exploration of the network scale traffic flow data can provide insights to the understanding and management of road network. While static bottlenecks have gained enough attention and many research results [3], such dynamic network bottleneck are still attracting ongoing researches.

Exploring network traffic congestion from traffic flow data is not a new idea. Due to the deployment of ITS, traffic flow data becomes more and more common nowadays. Various methods are proposed to achieve the task above. For example, Anbaroglu, Heydecker, and Cheng [4] used link journey time to cluster the congestion. The link was considered as congestion when the travel time exceeds a threshold; Hu et al. [5] used the vehicle trajectory data to identify the congestion along a road. The velocity at a moment was indicated by the gray pixel in the image. Then the congestion was identified by image processing method; Bauza and Gozalvez [6] proposed the congestion detection method based on vehicle-to-vehicle technology; Wang Lu et al. [7] visualized the traffic jam in Beijing using GPS trajectory data. The jam identification was realized by applying a speed threshold. And the data was assumed complete; Damaiyanti, Imawan, and Kwon [8] using a similar way to indicate the congestion as a heat map style. Due to large size of the data, map reduce framework was implemented. Another type of method is based on kernel density estimation (KDE) method. Li et al. [9] used FCD (floating car data) to estimate the kernel density of accessibility of POI (points of interest). The accessible cell was confined by the travel time and the predefined bandwidth. Schrank, Lomax, and Crum [10] developed a method to identify the Worst Bottlenecks in Texas State. The method used traffic speed data, roadway geometry, and traffic counts to calculate congestion-related performance measures. Performance measures such as annual delay per mile, congestion cost, and the Travel Time Index are produced from this analysis and were used to rank the congested segments across. John, Eisele, and Schrank [11] developed a method that can group links with similar traffic state using speed data. Pairs of links speed plot was established to get the similarity between adjacent links, in which similar traffic operation will result in linear relationship. Xu, Yue, and Li [12] proposed a multi-dimension analysis framework of traffic congestion based on historical floating car data set. The congestion event was identified by some spatial and temporal threshold; Wang, He, Stenneth et al. [13] proposed a traffic congestion estimation framework by using Twitter as the data source. Common twitter users’ traffic information were selected by matching at least one term of the predefined congestion vocabularies. Another kind of method that needs to be noticed is data cube[14], where traffic flow data are organized into a cube shape, with spatial-temporal dimensions, therefore different levels of analysis can be applied.

### Conclusion of Review

Although various methods including simulation results [15] are proposed to identify, estimate and descript the evolution of traffic congestion, still one problem remains unanswered: how to identify the bottleneck of the surface street network or urban road network. The congestion itself is the result of bottleneck dynamics. Reconstruction of bottleneck requires dynamic traffic flow data, which cannot be in great time scales such as hourly data, nor in small scales such as seconds, where great computation efficiency will be a big problem. Even under this condition, still many difficulties exist that can not be passed by. A typical example is the data missing problem. Traditionally the method either ignores the data, or supplements the missing part using average and similar data. It may create more errors. Moreover, the missing data may not just a decrease of information. For example, when there is no data in the hot-spot of CBD area, one possibility is that the congestion is so severe that no floating car can enter.

The purpose of this research is to answer the above question by a time series similarity measure approach. The idea is straightforward: the traffic data can be seen as many time series. And for those links in the same congestion area, the trend should be similar, i.e. the velocity decrease or restore at the similar pace. Strangely such characteristic is neglected in many researches, where just the threshold is applied to define the congestion.

The whole process of bottlenecks identification includes mainly two components:

- A time series similarity measure component, which is used to deal with the data missing problem;
- A clustering algorithm component, which identifies the spatial-temporal congestion by a metaheuristic way. The clustering is implemented in three levels, from homogeneous traffic state to single step heterogeneous condition, then to multi-steps heterogeneous condition.

**Fig 1:**
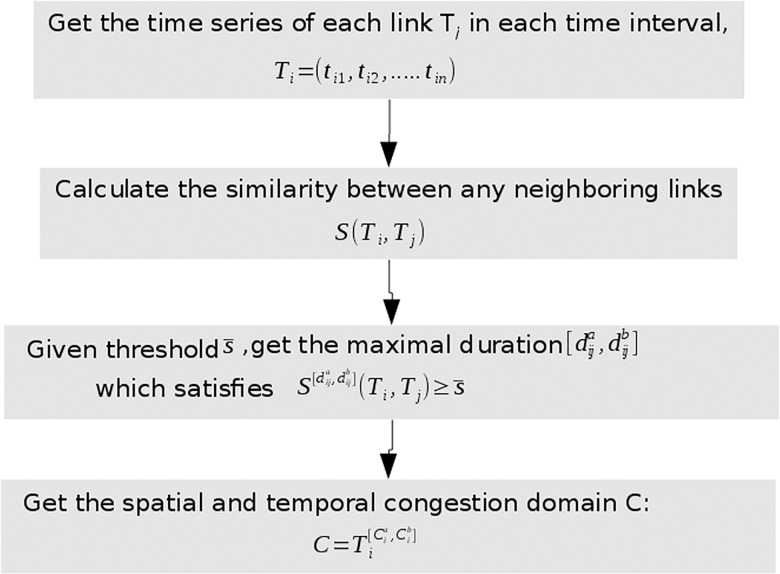
Flow chart of the method.

The following chart of the method is as in **Fig** 1:

## Notations

The notations are listed in Table 1.

**Table 1:** Notations list.

*Θ*_*i*_ = {*Θ*_*ij*_}	time series of link i at moment j. some values may be not available either because facilities failure or other factors, and thus *Θ*_*ij*_ = *NaN*, NaN means “not a number”;
${\bar{\theta }}_{i}$ and $\underline{\theta_{i}}$	Up-bound and down-bound limit of the reference index of link, such as velocity;
$s^{\left[t_{1},t_{2}\right]}(\theta_{i},\theta_{j})$	the similarity between time series *θ*_*i*_ and *θ*_*j*_ during the time domain [t_1_,t_2_] When the super script [t_1_,t_2_] is omitted, it means that the similarity is applied along the whole available time domain, i.e. s(*θ*_*i*,_*θ*_*j*_).
$\underline{s}$	predefined minimum threshold used in jam clustering.
*s*_*normal*_(*θ*_*i*,_*θ*_*j*_)	the similarity measure when the data are not missing. Under this case various measurement can be applied, such as cosine similarity, common Eculide distance etc.
*σ*_*ij*_	the adjacent matrix of the road network. When the head of link *i* and the tail of link *j* is the same, then *σ*_*ij*_ = 1, otherwise *σ*_*ij*_ = 0.
*d*_*ij*_	Distance matrix of the network. When the pair is not accessible, the respective value is infinite.
J = {*J*_*m*_}	J means the jam clustering within the whole temporal-spatial domain. And consists of all jam clusters within time interval or a specific time domain *m*. If at an interval *m* there is no jammed link, then J_m_ = ⌀. Otherwise *J*_*m*_ is defined as follows:
*J*_*m*_ = {*J*_*mk*_} and *J*_*mk*_ = {*θ*_*i*_}	*J*_*mk*_ means k-th jam cluster in time domain *m*. And each *J*_*mk*_ is a bottleneck area which consists of many links *θ*. These links form a weak connected sub-network of the whole network.
$L_{J_{m}}$	Overall length of jam cluster *J*_*m*_. It is the sum of all links within the cluster, i.e. $L_{J_{m}}=\sum_{i\in J_{m}}L_{\theta_{i}}$, where $L_{\theta_{i}}$ is the length of link *i*.
$N_{J_{m}}$	links number in jam bottleneck area. The clustering of jam clusters for figurative single jam cluster is formulated as $\max_{N_{J_{m}}}S^{m}(\theta_{i},\theta_{j})>\underline{s},\forall \theta_{i},\theta_{j}\in J_{m}$.
*T* and Δ*T*	T means the time domain for time similarity measure calculation, during which traffic state is considered as uniform across space. And Δ*T* means the rolling time step.

## Time Series Similarity Considering Missing Data

### The Model

Suppose we have two time series, *θ*_1_ = {*θ*_11_,*θ*_12_,*θ*_13_……*θ*_1*n*_}, and *θ*_2_ = {*θ*_21_,*θ*_22_,*θ*_23_……*θ*_2*n*_}. both series may have some missing data due to some factors. The value of missing data is expressed as NaN, which means “Not a Number”. Our objective is to calculate the similarity between *θ*_1_ and *θ*_2_:

$$S(\theta_{1},\;\theta_{2})$$ (1)

When there is no missing data, a variety of methods can be applied, such as cosine similarity, Euclid distance similarity, etc. We define the similarity under this case as normal similarity:

$$S_{normal}(\theta_{1},\;\theta_{2})$$ (2)

Actually there are many methods that can deal with missing data, for example we can just omit the missing part of a series (the corresponding data which is not missing in another series is omitted either); or supplement the missing data with for example the average across all possible values. Also we can use DTW (dynamic time wrapping) to deal with the two series with different length. However such method neglect the information behind the missing data. For example in the data set used where the data source is taxi GPS, the missing data may due to the severe congestion which impede the taxi to enter the link, or due to the low demand of the link/area. Simply drop the data or supplement it with mean may lose the underlying information and create great error.

Here we develop a method that can deal with missing data type time series similarity measurement. Since traffic parameters have its own limits such as velocity limit, we denote the up bound as $\bar{t}$ and down bound as $\underline{t}$. It means that any value in the time series are alway greater than $\underline{t}$ and smaller than $\bar{t}$.

In order to get the similarity measure between two time series, *θ*_1_ and *θ*_2_, both with the length K, with different number of missing data, we carry out the following process:

1. Abstract the elements in the original time series in pairs, for example we can abstract (*θ*_1*m*_,*θ*_1*n*_) and (*θ*_2*m*_,*θ*_2*n*_) for all m and n that satisfy *m* ≠ *n*. Thus there are $C_{K}^{2}=\frac{K(K-1)}{2}$ pairs. *K* is the length of the series.
2. With respect to each pair, (*θ*_1*m*_,*θ*_1*n*_) and (*θ*_2*m*_,*θ*_2*n*_), calculate the similarity measure *s*′_*mn*_ = s′((*θ*_1*m*_,*θ*_1*n*_),(*θ*_2*m*_,*θ*_2*n*_)) of this pair based on the following five cases. Let *p*_1_ = (*θ*_1*m*_,*θ*_1*n*_) and *p*_2_ = (*θ*_2*m*_,*θ*_2*n*_). The similarities are all expressed as an interval:
  - Case 1: All the data in the pair are not missing, then the similarity is:
    $${s\prime }_{mn}=s^{\prime }((\theta_{1m},\theta_{1n}),(\theta_{2m},\theta_{2n}))=s_{normal}((\theta_{1m},\theta_{1n}),(\theta_{2m},\theta_{2n}))$$ (3)
  - Case 2: Only one element in this pair is missing. Under this condition, there are mainly two cases: *θ*_1*m*_ = *NaN* or *θ*_1*n*_ = *NaN*. The similarity is calculated as follows:
    - Case 2.1 *θ*_1*m*_ = *NaN*
      We construct the vector $\bar{p_{1}}=(\bar{\theta_{1}},\theta_{1n})$ and $\underline{p_{1}}=(\underline{\theta_{1}},\theta_{1n})$. The up line and down line mean the up-bound and down-bound of the data. And since *θ*_1*m*_ = *NaN*, then the possible similarity between the pair is a interval, or a vector, not a scalar.
      In order to get the interval, firstly suppose *e* = (1,0), which is considered as unit vector. And then based on the simple geometry, the angle between vector *p*_1_ and the x-axis is between the interval of two angles:
      $$\bar{A_{1}}=arccos\left(\frac{e∙\bar{p_{1}}}{\left|\left|e\right||\;||\bar{p_{1}}|\right|}\right)$$ (4)
      $$\underline{A_{1}}=arccos\left(\frac{e∙\underline{p_{1}}}{\left|\left|e\right||\;||\underline{p_{1}}|\right|}\right)$$ (5)
      And the angle between vector *p*_2_ and the x-axis is:
      $$A_{2}=arccos\left(\frac{e∙p_{2}}{\left|\left|e\right||\;||p_{2}|\right|}\right)$$ (6)
      If $(A_{2}-\bar{A_{1}})(A_{2}-\underline{A_{1}})\leq 0$ it means that the vector *A*_2_ is between the vector $\bar{A_{1}}$ and $\underline{A_{1}}$, which makes the sign of $A_{2}-\bar{A_{1}}$ and $A_{2}-\underline{A_{1}}$ opposite, thus the maximum possible cosine similarity is 1 (the two series overlap) and the minimal similarity possibility is min$\left\{\cos(A_{2}-\bar{A_{1}}),\;\cos\left(A_{2}-\underline{A_{1}}\right)\right\}$. And if $(A_{2}-\bar{A_{1}})(A_{2}-\underline{A_{1}})>0$, it means that vector *A*_2_ lie outside the vector $\bar{A_{1}}$ and $\underline{A_{1}}$, and the minimal and maximal possible values of similarity are $\min\left\{\cos(A_{2}-\bar{A_{1}}),\;\cos\left(A_{2}-\underline{A_{1}}\right)\right\}$ and $\max\left\{\cos(A_{2}-\bar{A_{1}}),\;\cos\left(A_{2}-\underline{A_{1}}\right)\right\}$ respectively. Therefore we get the cosine similarity interval in (7).:
      $${s\prime }_{mn}=\left\{ \begin{array}{l} {}[min\{\cos(A_{2}-\bar{A_{1}}),\;\cos\left(A_{2}-\underline{A_{1}}\right)\},1]\;\mathrm{if}\;(A_{2}-\bar{A_{1}})(A_{2}-\underline{A_{1}})\leq 0\;\\ \;[\min\left\{\cos(A_{2}-\bar{A_{1}}),\;\cos\left(A_{2}-\underline{A_{1}}\right)\right\},max\{\cos(A_{2}-\bar{A_{1}}),\;\cos\left(A_{2}-\underline{A_{1}}\right)\}],\;otherwise \end{array}\right.$$ (7)
      $${s\prime }_{mn}=\left\{ \begin{array}{l} \;[min\;\{\cos\;(A_{2}-\bar{A_{1}}),\;\cos\;\left(A_{2}-\underline{A_{1}}\right)\},1],\;\mathrm{if}\;(A_{2}-\bar{A_{1}})(A_{2}-\underline{A_{1}})\leq 0\;\\ \;[\min\;\{\cos(A_{2}-\bar{A_{1}}),\;\cos\;\left(A_{2}-\underline{A_{1}}\right)\},\;\\ \max\;\{\cos(A_{2}-\bar{A_{1}}),\;\cos\;\left(A_{2}-\underline{A_{1}}\right)\}],\; \end{array}\right.$$
      Surely we can also use other types of similarity measure instead of cosine similarity. For example we can examine the Euclid distance between the fixed point *p*_2_ and the line between point $\bar{p_{1}}$ and point $\underline{p_{2}}$, but the basic principle is the same.
      The underlying idea of above equation is shown in the following **Fig** 2.

**Fig 2:**
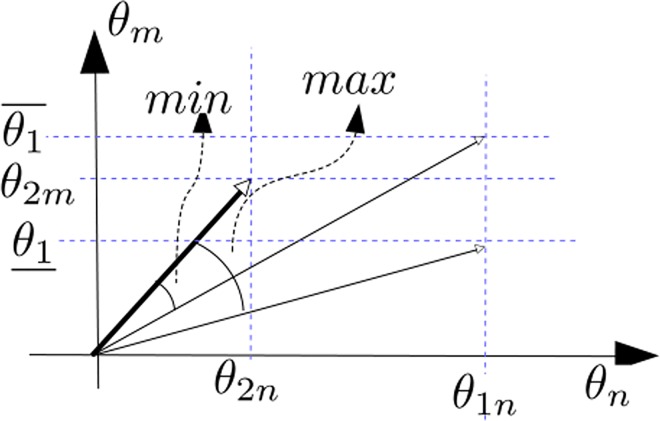
Underlying idea of similarity.
    - Case 2.2 *θ*_1*n*_ = *NaN*
      With respect to this type of missing data, it is similar to case 3.1. We just need to interchange the axis of ‘x’ and ‘y’ and construct another pair of *p*_1_ and *p*_2_. The formulation of (7) is also applicable.
  - Case 3 There are two missing data in the pair. Under this case, there are mainly three types:
    - Case 3.1 *θ*_1*m*_ = *NaN* and *θ*_1*n*_ = *NaN*
      Under this case the encompassing vectors that contain the vector *p*_1_ are $\bar{p_{1}}=[\bar{\theta },\underline{\theta }]$ and $\underline{p_{1}}=[\underline{\theta },\bar{\theta }]$. Thus we get the interval of its angles:
      $$\bar{A_{1}}=\arccos\left(\frac{e∙\bar{p_{1}}}{\left|\left|e\right||\;||\bar{p_{1}}|\right|}\right)$$ (8)
      $$\underline{A_{1}}=\arccos\left(\frac{e∙\underline{p_{1}}}{\left|\left|e\right||\;||\underline{p_{1}}|\right|}\right)$$ (9)
      And the angle of vector *p*_2_ is the same in Eq (6).
      The similarity measure under this condition is the same in (7).
    - Case 3.2 *θ*_1*n*_ = *NaN* and *θ*_2*m*_ = *NaN*
      Under this condition, both vectors lose one data point. Let $\underline{p_{1}}=(\theta_{1m},\underline{\theta_{1n}})$ and $\bar{p_{1}}=(\theta_{1m}\bar{\theta_{1n}})$. Similary we have $\underline{p_{2}}=(\theta_{2m},\underline{\theta_{2n}})$ and $\bar{p_{2}}=(\theta_{2m},\bar{\theta_{2n}})$. And we can get the interval between the angle formed by vectors and x-axis as $\left[\underline{A_{1}},\bar{A_{1}}\right]$ and $\left[\underline{A_{2}},\bar{A_{2}}\right]$, where:
      $$\left\{ \begin{array}{l} \bar{A_{1}}=\arccos\left(\frac{e∙\bar{p_{1}}}{\left|\left|e\right||\;||\bar{p_{1}}|\right|}\right)\\ \underline{A_{1}}=\arccos\left(\frac{e∙\underline{p_{1}}}{\left|\left|e\right||\;||\underline{p_{1}}|\right|}\right)\\ \bar{A_{2}}=\arccos\left(\frac{e∙\bar{p_{2}}}{\left|\left|e\right||\;||\bar{p_{2}}|\right|}\right)\\ \underline{A_{2}}=\arccos\left(\frac{e∙\underline{p_{2}}}{\left|\left|e\right||\;||\underline{p_{2}}|\right|}\right) \end{array}\right.$$ (10)
      If there is an overlap between $\left[\underline{A_{1}},\bar{A_{1}}\right]$ and $\left[\underline{A_{2}},\bar{A_{2}}\right]$, i.e. $\min\;\left(\bar{A_{1}},\bar{A_{2}}\right)\geq \max\;\left(\underline{A_{1}},\underline{A_{2}}\right)$, the maximum similarity would be one. The maximum angle between the two vectors is $\max\;(\bar{A_{1}},\bar{A_{2}})-\min\;\left(\underline{A_{1}},\underline{A_{2}}\right)$ and the minimum angle between the pair is:
      $$\max\;\left(\max\;(\bar{A_{1}},\bar{A_{2}})-\min\;\left(\underline{A_{1}},\underline{A_{2}}\right)-(\bar{A_{1}}-\underline{A_{1}})-(\bar{A_{2}}-\underline{A_{2}}),0\right)$$ (11)
      Therefore we get the similarity interval:
      $$\left[\cos(max(\bar{A_{1}},\bar{A_{2}})-\min\;(\underline{A_{1}},\underline{A_{2}})),\;\cos\left(max\left(max(\bar{A_{1}},\bar{A_{2}})-\min\;\left(\underline{A_{1}},\underline{A_{2}}\right)-(\bar{A_{1}}-\underline{A_{1}})-(\bar{A_{2}}-\underline{A_{2}}),0\right)\right)\right]$$ (12)
    - Case 3.3 *θ*_1*n*_ = *NaN* and *θ*_2*m*_ = *NaN*
      Under this condition, we can also construct the minimum and maximum angle between vectors. Thus Eq (12) can also apply, as long as the variables within the equation are replaced.
  - Case 4. There are three missing data. And without lose generality, we assume that *θ*_1*m*_ ≠ *NaN*
    Based on the derivations above, the related variable can be formulated as:
    $$\left\{ \begin{array}{c} \bar{p_{1}}=(\theta_{1m},\bar{\theta_{1}})\\ \underline{p_{1}}=(\theta_{1m},\underline{\theta_{1}})\\ \bar{p_{2}}=(\bar{\theta_{2}},\underline{\theta_{2}})\\ \underline{p_{2}}=(\underline{\theta_{2}},\theta_{2}) \end{array}\right.$$ (13)
    And:
    $$\left\{ \begin{array}{c} \bar{A_{1}}=\arccos\left(\frac{e∙\bar{p_{1}}}{\left|\left|e\right||\;||\bar{p_{1}}|\right|}\right)\\ \underline{A_{1}}=\arccos\left(\frac{e∙\underline{p_{1}}}{\left|\left|e\right||\;||\underline{p_{1}}|\right|}\right)\\ \bar{A_{2}}=\arccos\left(\frac{e∙\bar{p_{2}}}{\left|\left|e\right||\;||\bar{p_{2}}|\right|}\right)\\ \underline{A_{2}}=\arccos\left(\frac{e∙\underline{p_{2}}}{\left|\left|e\right||\;||\underline{p_{2}}|\right|}\right) \end{array}\right.$$ (14)
    The similarity measure of Eq (12) can either apply.
  - Case 5: All data are missing, i.e. (*θ*_1*m*_,*θ*_1*n*_) = (*NaN*,*NaN*) and (*θ*_2*m*_,*θ*_2*n*_) = (*NaN*,*NaN*).
    $$\left\{ \begin{array}{c} \bar{p_{1}}=(\bar{\theta_{1}},\underline{\theta_{1}})\\ \underline{p_{1}}=(\underline{\theta_{1}},\theta_{1})\\ \bar{p_{2}}=(\bar{\theta_{2}},\underline{\theta_{2}})\\ \underline{p_{2}}=(\underline{\theta_{2}},\theta_{2}) \end{array}\right.$$ (15)
    Similarity $\underline{A_{1}},\underline{A_{2}},\bar{A_{1}},\bar{A_{2}}$ are derived.
    The similarity is the same as in Eq (12)
    Since we will carry out similarity based on the intervals above, we call these intervals as first-order similarity.
3. In case 1 where no data are missing, we set *S*′_*mn*_ = [*S*_*normal*_((*θ*_1*m*_,*θ*_1*n*_),(*θ*_2*m*_,*θ*_2*n*_)),*S*_*normal*_((*θ*_1*m*_,*θ*_1*n*_),(*θ*_2*m*_,*θ*_2*n*_))].
  After the above process, we get $C_{K}^{2}=\frac{K(K-1)}{2}$ first order similarity measurements; each is a two variable vector thus can be described uniformly as $S_{mn}=\{[s_{mn}^{a},s_{mn}^{b}]\}$. Two time series from them then are constructed:
  $$s_{mn}^{a}=\{s_{11}^{a},s_{12}^{a},s_{13}^{a}\ldots \ldots \}$$ (16)
  $$s_{mn}^{b}=\{s_{11}^{b},s_{12}^{b},s_{13}^{b}\ldots \ldots \}$$

There is no missing data in the interval series. Many similarity measures can be applied to the intervals series. We call this the second-order similarity measures:

$$S(T_{1},T_{2})=s_{normal}(s_{mn}^{a},s_{mn}^{b})$$ (17)

### An Example of Missing Data Similarity

**Fig 3:**
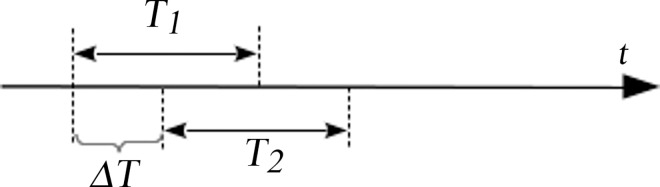
Time rolling horizon.

We randomly choose two adjacent links to give a presentation of the similarity measurement results. Note that we can set a time domain where the similarity measure is taken. When the similarity measurement is obtained, we move the time domain forward as in **Fig** 3 in a rolling horizon manner. In this way the variation of time series can be observed. The step and time domain length can both be changed.

**Fig 4:**
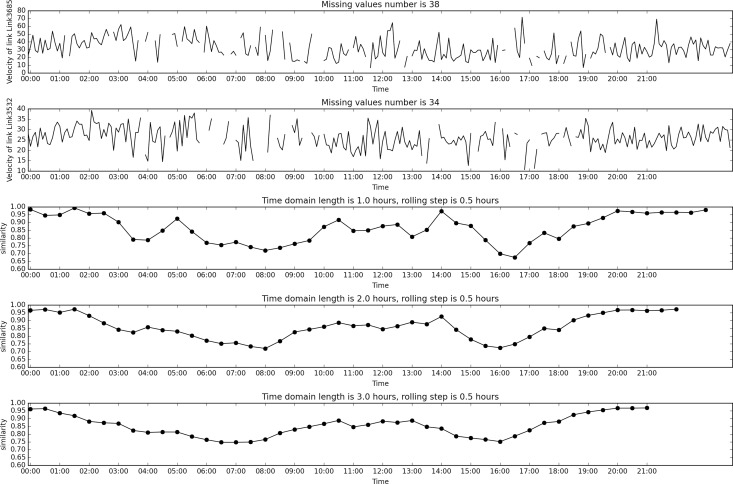
Similarity measure example.

**Fig** 4 shows the results for different time domain length. For both links there are some missing data. The missing data number for the first link is 38 and that for the second link is 34. There are also some features of gathering phenomena of the data, i.e. the data missing moments are relative close, mainly at peak hours, around about 06:00~07:00 and 16:00~17:00. The up bound and down bound of velocity is set based on the rank of the road. The major arterial road is set to 60km/h. Speed limit for minor arterial road and major branch are set to 50km/h and 40km/h respectively. Down bound of the velocity is set to zero.

The figures give three different time domain length, 1, 2 and 3 hours respectively. Rolling steps are all set to 0.5 hour. The velocity itself displays an irregular nature. From the trend of the velocity curve it is hard to decide at what moment is peak hour and at what moment is off-peak hour. But the missing data points appear mostly at peak hour based on the intuitive observation. This may be due to the congestion which prevents the vehicles from entering the links. When there is missing data, the resulting similarity is smaller, since the possible interval of the velocity changes from down bound to up bound. This can be seen from the similarity when time domain length is 1 hour. The lower similarity corresponds to the peak hour, when the velocity of the road network is very low (according to the last but one section where velocity profile of the whole network is presented). Because the time domain length is 1 hour thus the similarity for the last hour (23:00~24:00) cannot be calculated since there is no enough data. When the time domain length increases to 2 hours and 3 hours, the similarity measurements can also be calculated. The whole trend still holds, but the similarity measurement is smoothed. The longer the time domain is, the smoother the similarity is.

## Jam Clustering Method

A congestion event which covers many links will generate many similar time series. Vice versa, through the similarity between time series, we can reproduce the congestion profile based on traffic flow operational data. And during off-peak hours, random factors prevail more than peak hours. Thus similarity between peak hours is greater than that of off-peak hours. Given a similarity threshold, the area which shares similarity above the threshold can be sketched out. Combined with the operational data, the profile of a congestion can be derived.

### General Model

Clustering is based on the similarity measures between neighboring links. Generally, the links within the same congestion area are similar in distribution thus leads to a greater similarity measure. Traditional clustering methods cluster all the objects into finite clusters. No one is left outside the clusters. And the core of the clustering algorithm is distance measurement, or similarity measurement between each pair of objects. However this traditional clustering framework is not suitable for our problem in the following aspects:

- Not all of the objects, or links need to be clustered. Some links with very good traffic state don't belong to any congestion areas and due to their traffic performance no cluster requirement is needed. Mathematically clustering can be implemented on such links but the results make no sense;
- During the traditional clustering process, the similarity measure of an object with a cluster is either by measure the object with a representative of the cluster or with the ‘mean’ of this cluster. Such definition is easy to understand. However it is hard to decide or define which one is representative of the congestion area or the mean of the congestion area.

However, some principles of traditional clustering algorithm can still be applied. Define $\bar{J_{m}}$ as the subgraph that contains all links which don't belong to subgraph *J*_*m*_. For a jam cluster within a network, the definitions of a congestion cluster can be summarized as the following two conditions:

1. Time series *θ*_*i*_ of any link *i* within *J*_*m*_ is very similar to the whole jam cluster *J*_*m*_;
2. Time series *θ*_*i*_ of any links *i* outside *J*_*m*_ is distinctly different from the state of jam cluster *J*_*m*_;

In order to get the jam cluster *J*_*m*_, a similarity threshold $\underline{s}$ is set, the state above which is considered as congested.

Based on the above considerations, rather than using the traditional clustering framework, we introduce a metaheuristic algorithm for our congestion area identification. This algorithm first sets a similarity threshold, and then chooses the two links with the maximum similarity as a congestion core, and gradually attaches the neighboring links to the core to form a new congestion are and then repeat the attachment process until there are no neighboring links that satisfy the similarity threshold.

There are three levels of clusters according to the homogeneous of the traffic states.

### Metaheuristic Algorithms

#### Single cluster

Suppose within a time domain, there is only one jam cluster in the road network. And the traffic states of all links within the jam are simultaneous, i.e. the traffic velocity decrease and restore back at the same trend. Thus the overall traffic state is uniform across the network.

Under such condition, the problem is typically a clustering problem and the clustered objects are all links. The similarity between objects is given by the models above. Suppose the jam cluster is denoted by *J*_*m*_ where *m* is a specific time domain within which the traffic state can be considered as uniform. And *J*_*m*_ can be expressed as *J*_*m*_ = {*θ*_*i*_} where link *i* is considered as a congested link. Add all links that belongs to cluster *J*_*m*_ form a weak-connected subgraph. Thus *J*_*m*_ is also a subgraph of the original directed network.

**Fig 5:**
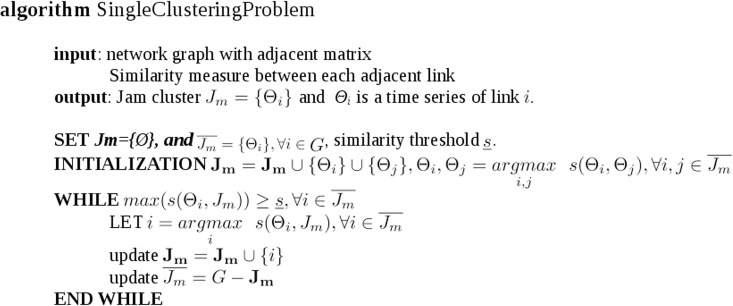
Pseudo code for single clustering.

The pseudo code for the single clustering problem can be formulated as in Fig 5:

#### Multiple simultaneous cluster in time interval

When there are some clusters at the same time and these congestion clusters do not overlap with each other, the above procedure will have some clusters left.

Since the clusters don't share some common links, when one cluster process is carried out, the same process can applied to the remained network again and get another cluster. Repeat the process until there is no cluster.

**Fig 6:**
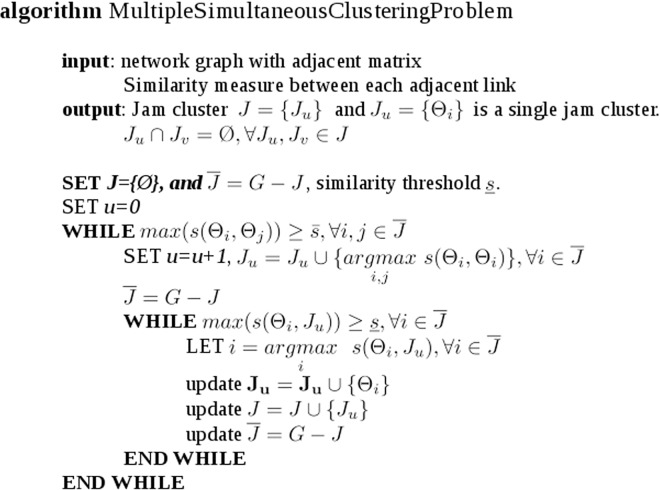
Pseudo code for Multiple clustering.

Therefore the pseudo code for the multiple clustering problem can be formulated as in **Fig** 6:

#### Multiple non-simultaneous cluster

Multiple non-simultaneous cluster means that these clusters do not take places at the same pace. For example some cluster may be at the time interval of 08:00~09:00 and others may be 08:30~09:15. In order to get this, the time horizon method is used. In the method, multiple Simultaneous Clustering process is used for one time domain, and the time domain rolls forward, and multiple Simultaneous Clustering process is applied again. Thus the jam clusters across the whole time horizon can be derived. Since the process is relatively simple, the pseudo code is omitted here.

## Field Tests of Model

This section applies the method above to HangZhou city, China to identify the dynamic bottleneck and presents the evaluation results of the network.

### Data Description

The data includes two parts: the geographical data set and the velocity data set. The geographical data is in a shape file which is developed and regulated by Esri and includes the detailed coordinates of each link. The adjacent relationship of all links also is covered. This data set helps to visualize the traffic state in the network. The other data is the average vehicle velocity data, which is the core of the paper. The raw data for calculating vehicle velocity is the taxi GPS data collected from 2012-11-01 to 2012-11-30 in city of Hangzhou, China. There are totally about 9000 taxis in the city at that time. The GPS equipment installed in each taxi sends second by second location and heading direction information to the traffic management center. The location can be matched to the geographical data to determine which link the taxi is driving on. In this way, the travel trajectory of each vehicle can be obtained. To calculate the velocity of each link, the entire modeling time horizon is divided into intervals of five-minutes. For a specific link *i* at time interval *k*, vehicles traversed the link is selected from raw data. Suppose that, for a vehicle selected within this time interval, the initial spatial-temporal coordinate is (*t*_1_,*x*_1_) and the final coordinate is (*t*_2_,*x*_2_), then the mean velocity within this spatial-temporal space can be calculated as $\frac{\left(x_{1},x_{2}\right)}{\left(t_{1},t_{2}\right)}$. These mean velocities are then averaged over all the vehicles to obtain the link velocity. For each link, there will be totally 288 successive velocities within an analysis period of 24 hours. The directional velocity is not considered. But for the description of the whole network, such simplification is acceptable. A two-way street is modeled as two links here, and the velocity is calculated separately for each link.

**Fig 7:**
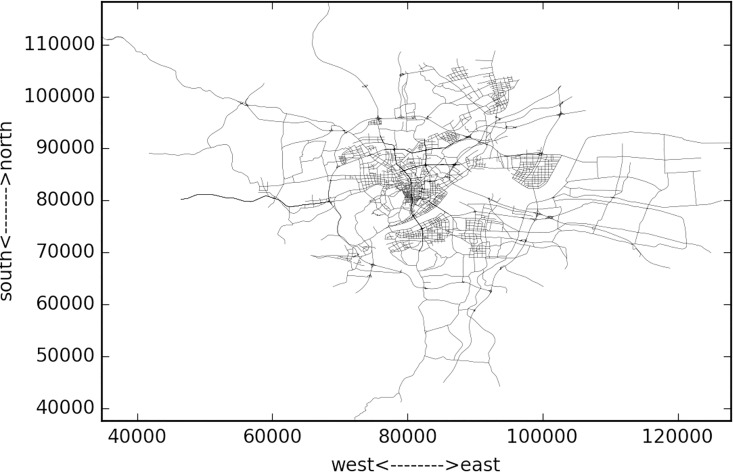
HangZhou road network.

**Fig 8:**
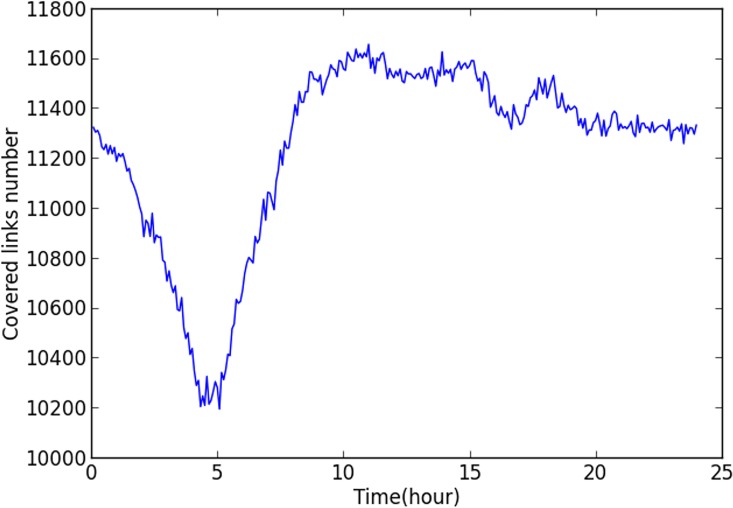
Data Completeness of HangZhou road network.

The studied network covers all the links within the “Ring Highway” of Hangzhou City, as shown in **Fig** 7. The horizontal and vertical boundary distances of the ring highway are about 30km and 35 km respectively. Within this area, there are about 13402 directional links. Taxis may not use some links during some time intervals, and **Fig** 8 shows the numbers of links covered by taxi trajectory during different times of a day. Since the majority of the roads velocity is available, and the number of links is relatively low in the early morning, and we mainly focus on the peak hours rather than off-peak hours, such coverage is acceptable.

**Fig 9:**
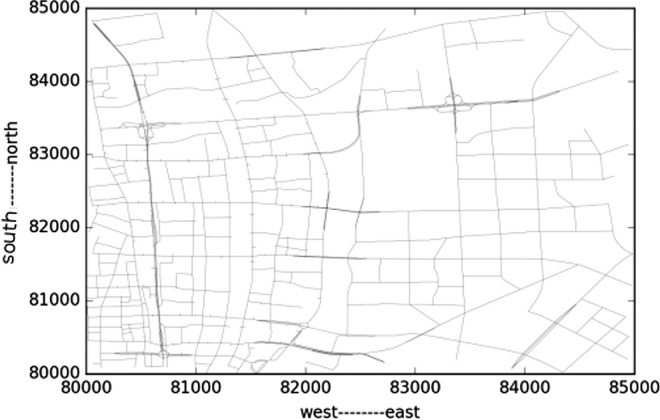
Sub-network used in the test.

Due to the huge computation issue, just part of the whole network is used. The sub-network is 5km*5km square area, as shown in **Fig** 9. In this area there are 2654 links, totally 5008 pair of adjacent links. For each link we get the velocity of one day and calculate the time rolling horizon similarity and cluster the jam areas based on the similarity measurement. The second order similarity is calculated using cosine similarity method. There data limit is based on velocity limit for each road rank. The ranks include principle arterial road, minor arterial road, principle branch, expressway and highway. The speed limits are as follows: principle arterial road limit is 50km/h; minor arterial road limit is 50km/h; principle branch road velocity limit is 40km/h; expressway limit is 80km/h; highway limit is 120km/h.

## Results

### Preliminary Results

We set the time domain length to 2 hours, which means the similarity is carried out with two hours. The rolling time step is either 2 hour. Therefore for a whole day, the clustering process is carried out 12 times. The congestion clusters within each time domain are generated.

**Fig 10:**
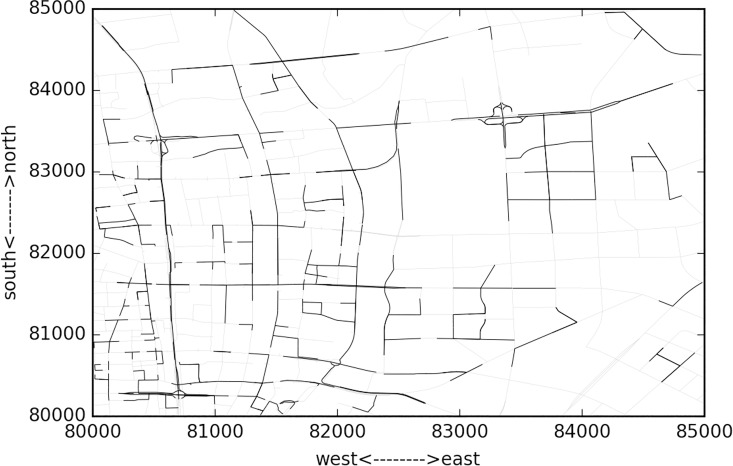
Cluster results at 08:00~10:00 time domain length = 2 hour and rolling time length = 2 hours.

**Fig 11:**
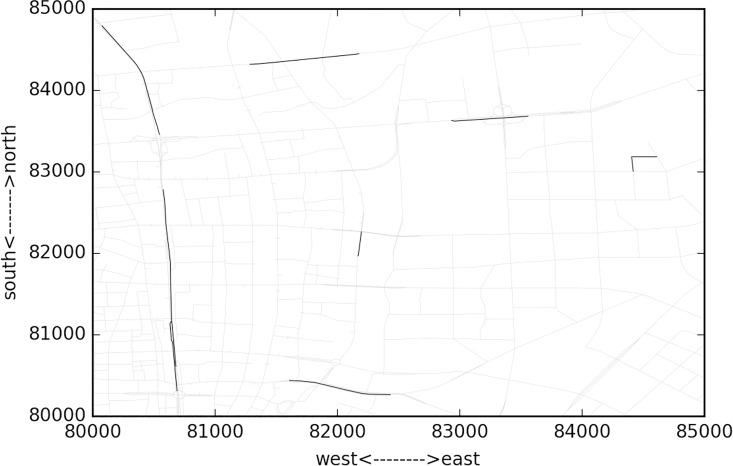
Cluster results at 02:00~04:00 time domain length = 2 hour and rolling time length = 2 hours.

**Fig 12:**
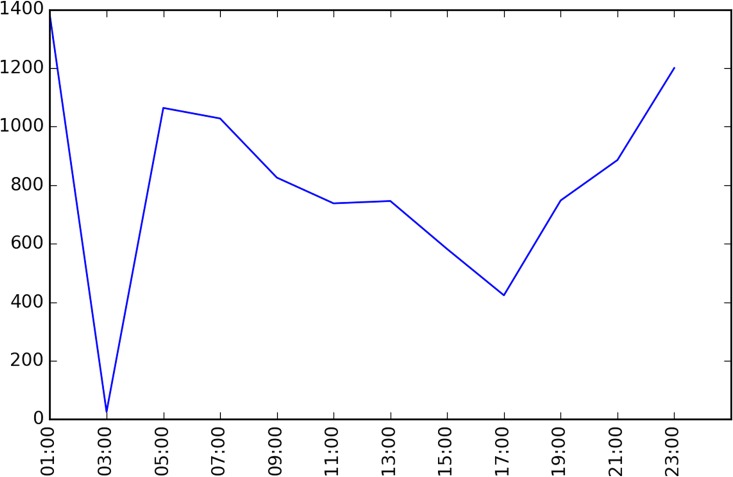
Size of the clusters along time.

**Fig** 10 presents the cluster at 08:00~10:00 and the similarity threshold is set as 0.99. From the figure, it can be observed that major clusters distribute along eastern-western and southern-northern arterials, typically the skyway, on-off ramps, intersections at major arterial roads. The covered links number occupies about 16% of the whole network. However, it is not the case for 02:00~04:00, when the morning traffic peak is not approaching yet. Fig 11 gives the results. It is shown that just the major skyway across the whole area and some major intersections is identified as congestion clusters. This is due to the fact that before the peak hour, some areas have relatively higher demand than other areas, typically the residential communities rather than the major roads. Due to the fact that most commuters choose the skyway as a route, it remains higher similarity even at this time domain. This can also be observed from Fig 12, where the number of clusters are presented along the time domains. At the first time domain, i.e. 00:00~02:00,due to the large scale missing data, many neighboring links both with many missing data points have a higher similarity, thus the number of links covered by the clusters are maximal. But it does not mean that the congestion is severe at that moment.

**Fig 13:**
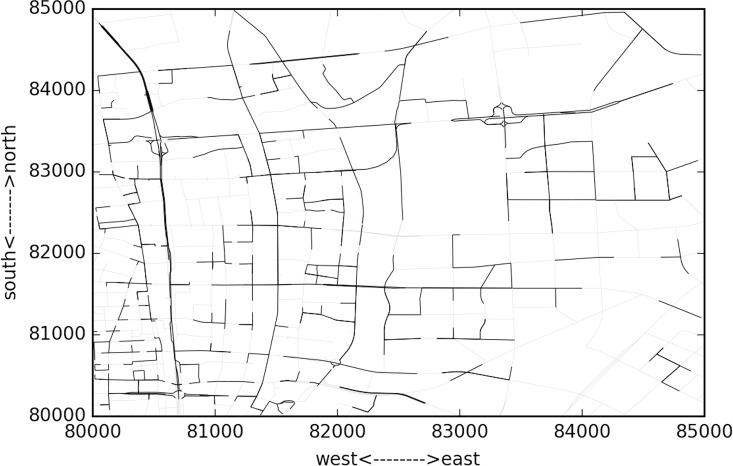
Cluster results for time domain length = 1.5 hour.

We change the time domain length to 1.5 hour and the rolling time step to 1.5 either to check the clustering result. **Fig** 13 gives the results for time from 07:30 to 09:00. All the clusters occupy 22% of the network, which is more than that of the same time period when the time domain length is 2 hours. Also the major arterial roads intersection and the skyway are identified as congestion clusters.

**Fig 14:**
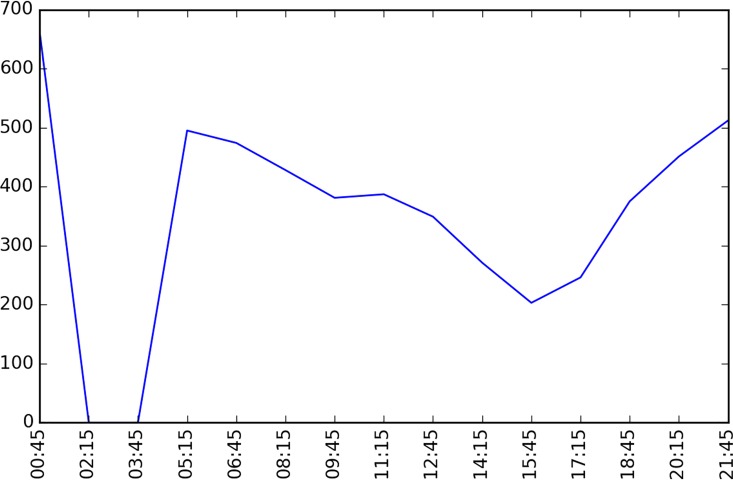
Common links number of neighboring time domains, time domain length = 1.5 hour.

For each time domain, many clusters of congestion links are identified. Some links may be identified as jam cluster in some domains while do not exist in other domains. Some links are always recognized as jam clusters. Thus the common links can be viewed as recurrent bottlenecks. **Fig** 14 displays the common links number along time. For the whole day, there is no common links that exist at all time domains. It indicates that there are no areas or links that are always congested. The trend of the common links number is very alike to **Fig** 12. In the deep night, due to the large scale loss of data, there are many links share higher similarities. When network traffic demands increase, the dis-similarity between areas makes it difficult to find a cluster. During the day time, the common links number firstly increases and then decreases.

#### Different threshold settings

**Fig 15:**
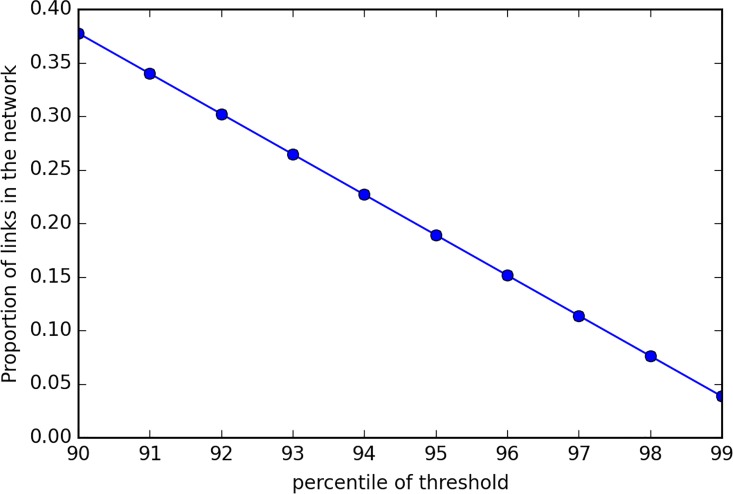
Proportion of links with Percentile.

Given different threshold of similarity in the process of clustering, the results would vary. When the threshold increases, the resulting clusters tend to be smaller. The cluster results under given threshold should be influenced by the distribution of the similarity across the whole network. The threshold can be given in two ways: either by giving a scalar or by the percentile of all similarities. Here the latter method is used. A percentile of all the similarities is set as a threshold and then the clustering process is implemented. When the percentile is 100%, then the resulting cluster would be zero since no similarity exceeds this threshold. When the threshold is set at 0%, the resulting cluster will be the whole network. **Fig** 15 presents the results applying different clustering threshold from the time domain 07:00 to 08:00. The proportion of links is calculated based on the target network, which contains 2654 links.

It seems that the size of clusters changes linearly with the percentile threshold. 90% percentile cluster occupy about 30% of the whole network, which implies that the network operates at a higher simultaneous manner.

## Conclusion

Traffic congestion on the road network involves both spatial and temporal variation. Information of when and where there is congestion will greatly benefit the following traffic flow operational state improvement. The task sometimes is difficult due to the missing data problem. This research deals with this problem using two stage measuring process. In the first stage the so called first order similarity is calculated given that the traffic flow variable is bounded at both sides. And the first order similarity is used to generate the second order similarity as the output. The spatial-temporal congestion is clustered in a heuristic manner based on the second-order similarity results.

Since the traffic flow data are collected network wide. If the network topology is considered, the accuracy of spatial-temporal congestion identification may be improved. For example, the shortest path, the feasible path set can be incorporated. And the similarity measurement may between unequal-length traffic flow data series. Another more important task is how to evaluate the congestion. Some indexes are possibly proposed to quantify the congestion, i.e. congestion quantum which is calculated by dividing congestion area by spatial-temporal resource.

Needless to say, such tasks are difficult. When suitable tools such as dynamic traffic assignment is combined, the interpretation and the further application of the method proposed is possible to be improved to achieve such tasks. These tasks will be next stage work.

## Supporting Information

S1 File Original map data and velocity data in the paper. (ZIP)
